# Myocardial electrophysiological and mechanical changes caused by moderate hypothermia—A clinical study

**DOI:** 10.14814/phy2.15259

**Published:** 2022-04-19

**Authors:** Kristin Wisløff‐Aase, Helge Skulstad, Kristina Haugaa, Per Snorre Lingaas, Jan Otto Beitnes, Per Steinar Halvorsen, Andreas Espinoza

**Affiliations:** ^1^ Institute of Clinical Medicine Faculty of Medicine University of Oslo Oslo Norway; ^2^ 155272 Departments of Research and Development Division of Emergencies and Critical Care Oslo University Hospital Oslo Norway; ^3^ 155272 ProCardio Centre for Innovation Department of Cardiology Oslo University Hospital Oslo Norway; ^4^ 155272 The Intervention Centre Oslo University Hospital Oslo Norway; ^5^ Karolinska Institute and Cardiovascular Division Faculty of Medicine Karolinska University Hospital Stockholm Sweden; ^6^ 155272 Department of Cardiothoracic Surgery Oslo University Hospital Oslo Norway

**Keywords:** dispersion of repolarization, electromechanical window, mechanical dispersion, moderate hypothermia, temperature control

## Abstract

Moderate hypothermia has been used to improve outcomes in comatose out‐of‐hospital cardiac arrest survivors during the past two decades, although the effects remain controversial. We have recently shown in an experimental study that myocardial electrophysiological and mechanical relationships were altered during moderate hypothermia. Electromechanical window positivity increased, and electrical dispersion of repolarization decreased, both of which are changes associated with decreased arrhythmogenicity in clinical conditions. Mechanical dispersion, a parameter also linked to arrhythmic risk, remained unaltered. Whether corresponding electrophysiological and mechanical changes occur in humans during moderate hypothermia, has not been previously explored. Twenty patients with normal left ventricular function were included. Measurements were obtained at 36 and 32°C prior to ascending aortic repair while on partial cardiopulmonary bypass and at 36°C after repair. Registrations were performed in the presence of both spontaneous and comparable paced heart rate during standardized loading conditions. The following electrical and mechanical parameters were explored: (1) Electromechanical window, measured as time difference between mechanical and electrical systole, (2) dispersion of repolarization from ECG T‐wave, and (3) mechanical dispersion, measured as segmental variation in time to peak echocardiographic strain. At moderate hypothermia, mechanical systolic prolongation (425 ± 43–588 ± 67 ms, *p* < 0.001) exceeded electrical systolic prolongation (397 ± 49–497 ± 79 ms, *p* < 0.001), whereby, electromechanical window positivity increased (29 ± 30–86 ± 50 ms, *p* < 0.001). Dispersion of repolarization and mechanical dispersion remained unchanged. Corresponding electrophysiological and mechanical relationships were present at comparable paced heart rates. After rewarming, the increased electromechanical window was reversed in the presence of both spontaneous and paced heart rates. Moderate hypothermia increased electromechanical window positivity, while dispersion of repolarization and mechanical dispersion remained unchanged. This impact of hypothermia may be clinically relevant for selected groups of patients after cardiac arrest.

## Graphical abstract

Moderate hypothermia stabilizes electromechanical relationships associated with arrhythmic susceptibility in clinical conditions.

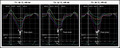

## 1 INTRODUCTION

Temperature control (32–36°C) has been used for neuroprotection in comatose cardiac arrest survivors over the last two decades (Nolan et al., 2015). The effects of therapeutic hypothermia are a matter of controversy, and the European resuscitation guidelines have recently been revised (Sandroni et al., 2022). Hypothermia prolongs electrical and mechanical systole (Emslie‐Smith et al., 1959; Lebiedz et al., 2012; Wisløff‐Aase et al., 2022). These electrical and mechanical events are closely linked. Changes in their relationships with altered durations or dispersed activity are associated with increased susceptibility to arrhythmia during normothermia in a variety of clinical conditions (Bekke et al., 2015; Leren et al., 2015; Watanabe et al., 2004). The risk of ventricular arrhythmia is increased at temperatures <32°C (Dietrichs et al., 2019). However, there is no evidence of increased ventricular arrhythmogenicity during temperature control at moderate hypothermia; that is, 32–34°C compared to 36°C (Lions et al., 2018; Nielsen et al., 2011), nor an association between increased QT interval and ventricular arrhythmic risk (Kim et al., 2014; Storm et al., 2011; Thomsen et al., 2021). In a recently published experimental *in vivo* study, we have demonstrated that moderate hypothermia induced changes in electrophysiological and mechanical relationships associated with reduced arrhythmogenicity in other clinical settings (Sugrue et al., 2020; Watanabe et al., 2004). The duration of electromechanical window positivity increased, and dispersion of repolarization decreased. Mechanical dispersion remained unchanged (Wisløff‐Aase et al., 2020). Whether corresponding temperature‐related changes occur in humans, has not previously been explored.

During normal physiological conditions, the electrical systole, that is, QT interval, ends immediately before the end of the mechanical systole (Boudoulas et al., 1982) giving a time difference denominated as positive electromechanical window (Linde et al., 2010). Electrical repolarization is only slightly dispersed throughout the myocardium (Opthof et al., 2016) and mechanical peak contractions in left ventricular segments occur with minimal dispersion in time (Verdugo‐Marchese et al., 2020). Prolongation of the QT interval is a risk factor for ventricular arrhythmias and cardiac arrest in the general population without cardiac dysfunction (Algra et al., 1991). In patients with diagnosed long QT syndrome, an even stronger correlation is shown if the QT interval outlasts the mechanical systole, leading to a negative electromechanical window (Sugrue et al., 2020). QT prolongation is also associated with increased electrical dispersion of repolarization and mechanical dispersion. Dispersion of repolarization and mechanical dispersion increase arrhythmic susceptibility as seen in long QT syndrome, hypertrophic cardiomyopathy, heart failure and after myocardial infarction (Haland et al., 2016; Hasselberg et al., 2016; Haugaa et al., 2010; Watanabe et al., 2004). At moderate hypothermia, QRS duration, QT interval, and systolic mechanical contraction are prolonged at spontaneous heart rate (HR; Lebiedz et al., 2012; Piktel et al., 2012; Rosol et al., 2017). The impact of moderate hypothermia on these adjacent electrophysiological and mechanical relationships in humans is unknown.

The aim of the present clinical study was to assess the isolated effects of moderate hypothermia on electrophysiological and mechanical relationships in human hearts without cardiac dysfunction and previous cardiac arrest, during standardized clinical conditions. The study had a unique design analogous to the experimental model applied in our previous animal study (Wisløff‐Aase et al., 2020). Based on the experimental results, we hypothesized that during moderate hypothermia at 32°C, left ventricle electrophysiological and mechanical conditions would lead to increased electromechanical window, but without any increase in electrical and mechanical dispersion.

## 2 METHODS

The study was designed to isolate the effects of temperature on myocardial electrophysiological and mechanical relations. The study protocol and effects on global left ventricular function have previously been reported (Wisløff‐Aase et al., 2022). Patients without evident cardiac dysfunction scheduled for elective ascending aortic surgery were included. To minimize heterogeneity in myocardial function and left ventricular loading conditions, exclusion criteria were ejection fraction <55%, previous myocardial infarction, atrial fibrillation, or planned aortic valve surgery. Written informed consent was obtained for all patients. The study was approved by the Regional Committee for Medical and Health Research Ethics, South‐East Norway (2013/565 B).

### 2.1 Anaesthesia, technical instrumentation, and surgical procedures

The patients were pre‐medicated with diazepam (5–10 mg). Anaesthesia was induced by intravenous (i.v.) fentanyl (3.5–7.5 μg/kg), midazolam (0.05–0.15 mg/kg), thiopental (2.5–7.0 mg/kg), and cisatracurium (0.15 mg/kg) and maintained with sevoflurane‐inhalation (1.0–2.5%) and repeated doses of iv. fentanyl (1–2 μg/kg). The patients were monitored according to standard clinical practice at our institution, in addition to the monitoring defined by the study protocol. Three‐lead electrocardiogram (ECG) was obtained by surface leads. Blood pressure was registered from a radial artery line. Central venous catheter (Arrow International Inc.), and pulmonary artery catheter (Swan‐Ganz CCO; Edwards Lifesciences Corporation) were inserted to obtain central venous pressure (CVP), and temperature and hemodynamic registrations, respectively. After induction of anesthesia, the patients underwent sternotomy and pericardiotomy, before atrial pacemaker leads (Medtronic streamline; Medtronic Inc.) were sutured on the right atrium. The patients were cannulated and connected to cardiopulmonary bypass (CPB), (Stöckert S5; Sarin Group Deutschland GmbH). During CPB, ventilation was discontinued, and sedation was provided with propofol infusion (3.5 mg/kg/h). All patients received thiopental (1 g) and methylprednisolone (2 g) prior to graft procedure. While the distal anastomosis was performed, the patients were cooled to deep hypothermia (<28°C). 18/20 patients underwent brief circulatory arrest in this phase. Myocardial preservation with ice cold cardioplegia inducing cold ischemia, was performed in all patients before suturing of the proximal anastomosis. After completing aortic repair and cardiac reperfusion, the patients were rewarmed and weaned off CPB.

### 2.2 Study protocol

**FIGURE 1:**
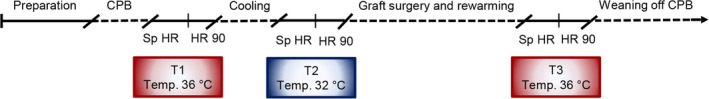
Timeline showing procedural sequence in relation to temperature and measurements at T1, T2 and T3. CPB, cardiopulmonary bypass; Sp HR, spontaneous heart rate; HR 90, atrial paced heart rate 90 beats per minute

All measurements were made while the patients were on cardiopulmonary bypass (CPB) to achieve optimal and uniform conditions. The measurements were made at three time points (Figure 1): T1 at 36°C, defined as baseline; T2 at 32°C prior to graft surgery, defined as moderate hypothermia; and T3 at rewarming to 36°C after aortic repair and cardiac reperfusion. As the body temperature decreased to <37°C during surgical preparations, 36°C was chosen as baseline temperature to avoid excessive time spent on rewarming to normothermia, and in accordance with the targeted temperature management recommendations in the current resuscitation guidelines. All recordings were made both during spontaneous HR, and at atrial paced HR of 90 beats per minute (bpm) to compensate for individual HR variability, and to adjust for the known effects of hypothermia‐induced bradycardia, both which could influence electrical and mechanical systolic duration. Atrial pacing was chosen to ensure electrical and mechanical activation of the left ventricle via the normal conduction route. Two patients had spontaneous HR ≥90 bpm at T1, and their data were excluded from the paced HR comparisons at that time point. One patient developed atrial fibrillation at 32°C; accordingly, the measurements at T2 for this patient were excluded, but registrations at T1 and T3 were included in the statistical comparisons.

The surgical setting, with use of CPB‐machine with a heat‐exchanger, enabled standardization with accurate control of body temperature and loading conditions. To obtain comparable and near‐normal cardiac working conditions, CPB flow was carefully reduced to 20% of the estimated maximum flow, hence ventilation was intermittently re‐continued. Loading conditions were made comparable by clamping venous drainage adjusted to mean arterial pressure (MAP) >50 mmHg and central venous pressure (CVP) ±10% of the baseline value. All measurements were performed during a stable phase, with no change in anesthesia or hemodynamic support. Surgical manipulation was paused during the measurements and between T1 and T2. Low dose norepinephrine (0.01 μg/kg/min) was used in two patients during T1 and T2, and low dose nitroprusside infusion (0.25–0.5 μg/kg/min) was continued in five patients at T3. These exceptions were controlled for and had no influence on statistical significance; thus data from all the 20 patients included are presented.

### 2.3 Transesophageal echocardiographic recordings

A Vivid E95 scanner (GE Vingmed Ultrasound) was used for echocardiographic 2D and Pulsed Wave (PW) Doppler recordings with a 5 MHz transesophageal echocardiographic probe (6VT‐D; GE Vingmed Ultrasound). The recordings were obtained from mid‐oesophageal two‐ and four‐chamber and long‐axis views, and transgastric short‐axis view at mid‐papillary level. All recordings were analyzed offline by designated software (EchoPac version 203; GE Healthcare). Measurements were made from three consecutive heart cycles and averaged. Four isolated measurements from segment 2 were foreshortened at T2 in three different patients at spontaneous heart rate; hence these measurements were excluded from the calculation of mechanical dispersion. Data were de‐identified, and the investigator was blinded to patient identification and situation.

### 2.4 Calculations of the electrical events

The standardized electrocardiogram (ECG) lead II, was used for electrical measurements and was synchronized with the echocardiographic scanner. QRS complex duration (QRS) was measured from onset of the Q wave to end of the S wave. Electrical systole was represented by QT interval and measured from ECG onset of QRS complex to end of T‐wave (Te), and this was also HR corrected (QTc = QT/√R−R interval; Bazett, 1920). The manual tangent method was used to determine Te, defined by the intersection of the isoelectric line with the tangent to the steepest downslope of the T‐wave (Postema et al., 2008). Dispersion of repolarization was measured from the ECG T‐wave as inter‐individual variation in duration of the T‐peak to T‐end interval (TpTe). T‐peak (Tp) was defined as the maximum absolute positive or negative deflection of the T‐wave from the isoelectric line (Rosenthal et al., 2018).TpTe was corrected for HR (TpTeC=TpTe/√R‐R interval).

### 2.5 Calculation of mechanical events

Aortic‐ and mitral valvular opening and closing were registered from echocardiographic recordings in long‐axis view. Ejection time was measured from aortic valve opening (AVO) to aortic valve closing (AVC). QAVC was measured from onset of the QRS to AVC. Isovolumic contraction time (IVCT) and isovolumic relaxation time (IVRT) were measured from mitral valve closing (MVC) to AVO and AVC to mitral valve opening (MVO), respectively. Diastolic filling time was measured from MVO to MVC. Mechanical systole was defined as QAVC. Electromechanical window was calculated as the difference between mechanical and electrical systole measured in the same beat (QAVC–QT).

### 2.6 Regional strain and mechanical dispersion

**FIGURE 2:**
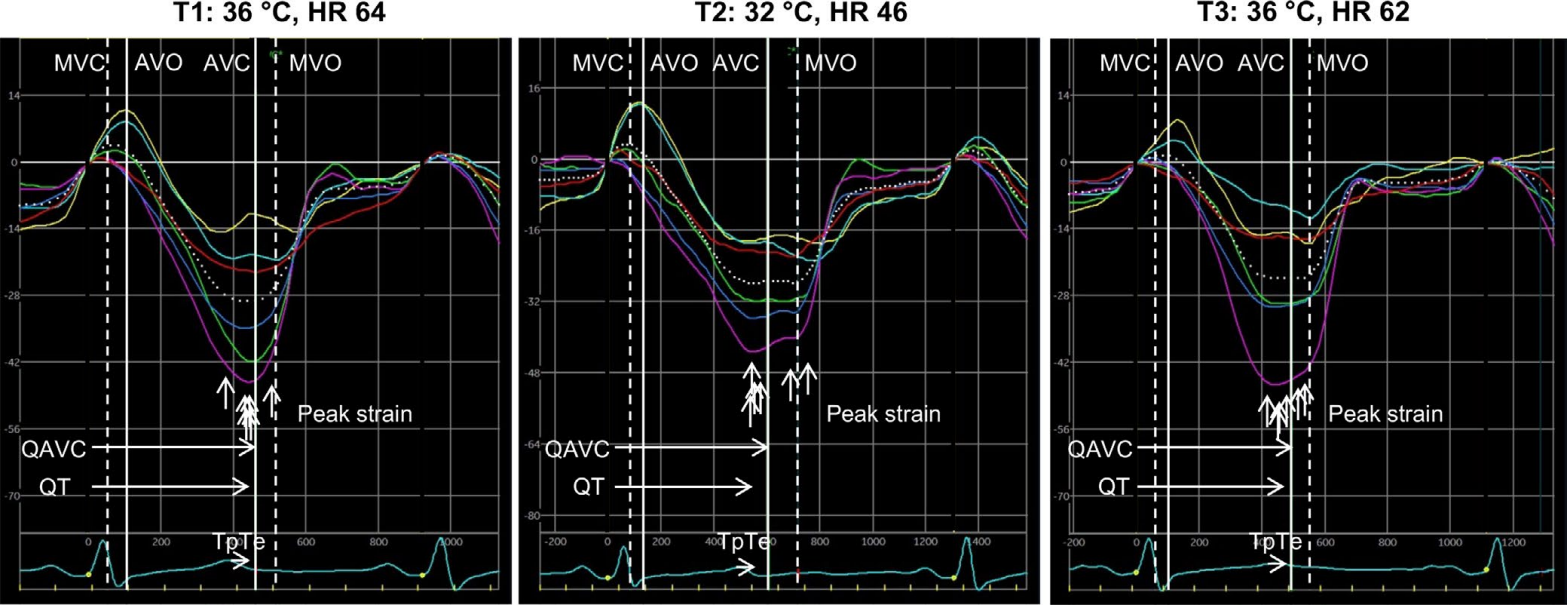
Longitudinal strain by speckle tracking echocardiography in apical long axis view at baseline: T1:36°C, at moderate hypothermia: T2:32°C and after rewarming: T3:36°C, at spontaneous heart rate (HR). White lines indicate aortic valve opening (AVO) and closing (AVC), and white dotted lines mitral valve opening (MVO) and closing (MVC). QAVC, QT‐interval and TpTe are marked. Electromechanical window is represented by the difference QAVC‐QT, dispersion of repolarization by the inter‐individual variance in TpTe, and mechanical dispersion as variation in time to peak strain, marked with white vertical arrows. Electromechanical window positivity is increased, while dispersion of repolarization and mechanical dispersion are unchanged at moderate hypothermia. Electromechanical window is returned to baseline value after rewarming

Speckle tracking echocardiography was used to obtain longitudinal strain in four‐chamber, two‐chamber, and long‐axis views (Edvardsen et al., 2002; Urheim et al., 2000) from 2D recordings with frame rate 52 ± 11 ms. The endocardial border was traced manually, and segments were adjusted to the myocardial thickness. Time to peak strain was defined from QRS‐onset in ECG to peak longitudinal strain in each segment, (Figure 2). Time to peak strain were measured from 18 segments. Mechanical dispersion was calculated as standard deviation of time to peak strain in the 18 segments model (Haugaa et al., 2010).

### 2.7 Statistical analyses

Sample size calculation was performed based on previous experimental results, to provide 80% statistical power to identify ≥20% change in global left ventricular function (SV and CI) with a two‐sided alpha level of 0.05. Statistical analyses were calculated in SPSS v.26 software (SPSS Inc.). Data are presented as mean ± standard deviation. One‐way analysis of variance (ANOVA) for repeated measures was used to determine whether there were any significant differences between the three time points with spontaneous HR, and between the time points with paced HR. Comparisons between time points T2 and T1, and T3 and T1, were performed by paired Student's *t*‐test. *p* value <0.05 was considered statistically significant.

## 3 RESULTS

Twenty patients were included (Table 1). Recordings of good quality were achieved at all‐time points at both spontaneous and paced heart rates. Moderate hypothermia (32°C) significantly reduced HR from 70 ± 14 to 53 ± 11 bpm, *p* < 0.001. Rewarming reversed these changes (65 ± 11 bpm, *p* = 0.079).

**TABLE 1:** Patient characteristics and general methodology

Variable	Value
Male/Female (*n*)	12/8
Age (year)	63 ± 14
Body mass index (kg/m^2^)	26 ± 3.3
Beta‐blockade (*n*)	8
Time per measurement (min)	7 ± 2
Time, cooling from T1; 36° to T2; 32° (min)	10 ± 3
Duration of hypothermia <36°C (min)	81 ± 24
Total cardiopulmonary bypass time (min)	127 ± 26
Cross‐clamp time (min)	48 ± 23
Surgery time (min)	181 ± 28

Note

Data expressed as mean ± SD and numerical (*n*).

### 3.1 Electrophysiological and mechanical changes at moderate hypothermia

**FIGURE 3:**
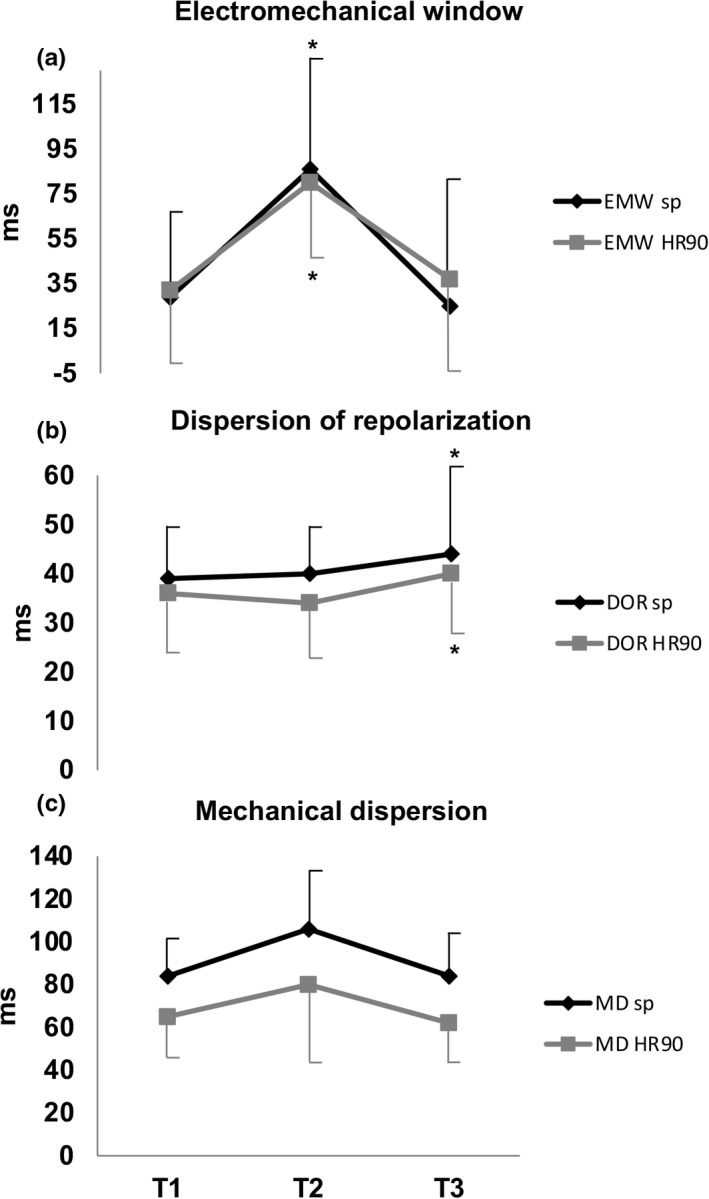
Electromechanical relations at baseline: T1:36°C, at moderate hypothermia: T2:32°C and after rewarming: T3:36°C, at spontaneous heart rate and heart rate 90 beats per minute, respectively. (a) Electromechanical window (EMW); (b) Dispersion of repolarization (DOR) and (c) Mechanical dispersion (MD). Mean values are presented with standard deviation. □ represents estimates and * denotes significant interaction between groups: T2 versus T1, and T3 versus T1

At baseline (T1; 36°C), electrical systole ended just before the end of mechanical systole, giving a positive electromechanical window. During moderate hypothermia (T2; 32°C) duration of the electrical and mechanical systole both increased (Table 2). The mechanical prolongation exceeded the electrical prolongation, such that the electromechanical window became more positive (Table 3; Figure 3). Dispersion of repolarization was not increased during moderate hypothermia. IVCT was unchanged but ejection time and time to peak strain increased. Mechanical dispersion remained unchanged. Electrophysiological and mechanical alterations during comparable paced HR of 90 bpm at T2, were consistent with the changes at spontaneous HR (Table 3; Figure 3).

**TABLE 2:** Electrical and mechanical parameters

Variable	Spontaneous heart rate			Heart rate 90 beats per minute		
	T1:36°C	T2:32°C	T3:36°C Post	T1:36°C	T2:32°C	T3:36°C post
Heart rate (bpm)	70 ± 14	53 ± 11	65 ± 11	90 ± 1.2	89 ± 1.3	89 ± 1.3
*p*		<0.001	0.079		0.616	0.571
R‐R interval (ms)	862 ± 170	1156 ± 254	937 ± 176	662 ± 29	669 ± 18	666 ± 5
*p*		<0.001	0.025		0.164	0.347
QRS‐complex (ms)	63 ± 4	68 ± 5	67 ± 9	62 ± 5	66 ± 5	65 ± 8
*p*		<0.001	0.006		<0.001	0.005
QT‐interval (ms)	397 ± 49	497 ± 79	429 ± 68	353 ± 31	391 ± 42	381 ± 36
*p*		<0.001	<0.001		<0.001	<0.001
QTc‐interval (ms)	431 ± 46	463 ± 45	449 ± 63	434 ± 39	478 ± 52	467 ± 44
*p*		<0.001	0.021		<0.001	<0.001
QAVC (ms)	425 ± 43	588 ± 67	465 ± 36	391 ± 33	470 ± 47	412 ± 41
*p*		<0.001	<0.001		<0.001	0.038
Isovolumic contraction time (ms)	46 ± 22	45 ± 16	43 ± 25	42 ± 21	44 ± 17	53 ± 26
*p*		0.603	0.862		0.560	0.363
Ejection time (ms)	299 ± 38	450 ± 71	326 ± 29	261 ± 35	320 ± 43	267 ± 42
*p*		<0.001	0.004		<0.001	0.304
Time to peak strain (ms)	395 ± 58	521 ± 90	421 ± 55	352 ± 52	390 ± 78	378 ± 51
*p*		<0.001	<0.002		<0.001	<0.001

Note

Data expressed as mean ± SD, *p* < 0.05 is considered significant. *p*‐value represents comparison between groups: at baseline T1:36°C versus moderate hypothermia T2:32°C, and after rewarming post‐surgery at T3:36 E°C versus baseline T1:36°C, at spontaneous and increased heart rate 90 beats per minute. T1 heart rate 90 beats per minute: *n* = 18, T2 spontaneous heart rate and heart rate 90 beats per minute: *n* = 19.

**TABLE 3:** Electrophysiological and mechanical relations

Variable	Spontaneous heart rate			Heart rate 90 beats per minute		
	T1:36°C	T2:32°C	T3:36°C post	T1:36°C	T2:32°C	T3:36°C post
Electromechanical window (ms)	29 ± 30	86 ± 50	25 ± 58	32 ± 34	80 ± 36	37 ± 42
*p*		<0.001	0.564		<0.001	0.705
Dispersion of repolarization (ms)	39 ± 10	40 ± 12	44 ± 19	36 ± 10	34 ± 10	40 ± 12
*p*		0.438	0.029		0.557	0.008
Mechanical dispersion (ms)	84 ± 26	106 ± 37	84 ± 28	65 ± 19	80 ± 41	62 ± 20
*p*		0.075	0.914		0.203	0.416

Note

Data expressed as mean ± SD, *p* < 0.05 is considered significant. *p*‐value represents comparison between groups: at baseline T1:36°C versus moderate hypothermia T2:32°C, and after rewarming post‐surgery at T3:36°C versus baseline T1:36°C, at spontaneous and increased heart rate 90 beats per minute. T1 heart rate 90 beats per minute: *n* = 18, T2 spontaneous heart rate and heart rate 90 beats per minute: *n* = 19.

### 3.2 Electrophysiological and mechanical changes after rewarming

After rewarming at T3 (36°C), both electrical and mechanical systole were still prolonged, compared to baseline at T1. However, the electromechanical window was not significantly different compared with the baseline value (Figure 3). Dispersion of repolarization was slightly increased compared to T1 (Figure 3). Mechanical dispersion remained unchanged (Figure 3). These findings were present also during comparable paced HR of 90 bpm.

## 4 DISCUSSION

This prospective clinical study is the first to explore the isolated effects of moderate hypothermia on electrophysiological and mechanical relationships in patients, by use of a study protocol approximating an experimental design. Electromechanical window positivity increased at 32°C compared to 36°C, while dispersion of repolarization and mechanical dispersion remained unchanged. The increased electromechanical window positivity was reversed to baseline values after rewarming.

The electromechanical window is a novel clinical parameter and has not previously been described during moderate hypothermia. In the present study, we found that the QT interval was extended beyond the normal range at moderate hypothermia (Viskin, 2009). Interestingly, the mechanical systolic prolongation exceeded this QT prolongation with a corresponding increase in electromechanical window positivity at 32°C, at spontaneous and comparable paced HR. These findings are consistent with earlier experimental results describing increased electromechanical window at temperatures <36°C (Guns et al., 2012; Wisløff‐Aase et al., 2020). The electromechanical window is experimentally shown to have better predictive power for ventricular arrhythmias than the QT duration, in ischemic heart disease (Limprasutr et al., 2018) and in patients with long QT syndrome (ter Bekke et al., 2015). The parameter is associated with increased arrhythmic risk at both negative and excessive positive values exceeding +111 ms in patients with channelopathies (Bekke et al., 2015; Schimpf et al., 2008). Whether a corresponding relation exists in other clinical conditions is unknown. However, clinical data strongly suggest that electromechanical window positivity is associated with reduced arrhythmic susceptibility (Bekke et al., 2015). Whether our finding represents an attenuation of a possible increased arrhythmic risk associated with the prolonged QT interval during moderate hypothermia, is not possible to conclude. Clinical studies evaluating electromechanical window and arrhythmic susceptibility during moderate hypothermia, are warranted to elucidate this relationship.

After rewarming, we found that both electrical and mechanical systole remained prolonged at spontaneous HR. The non‐significant trend toward decreased HR after rewarming may have influenced these measurements. Yet, prolonged electrical and mechanical systole were also present at comparable paced HR. These findings may represent a consequence of myocardial stunning due to deep hypothermia and cold ischemia during graft surgery. Importantly, the electromechanical interrelations were restored, and the electromechanical window returned to baseline values, indicating a rapid reversibility of the isolated effects of moderate hypothermia on these parameters.

We found no increase in dispersion of repolarization during moderate hypothermia, either at spontaneous or comparable HR. Dispersion of repolarization measured by TpTe has frequently been applied as a surrogate marker for heterogeneity in repolarization, although this has been controversial (Opthof et al., 2016). Arrhythmogenic gradients in repolarization may occur in the heart without changes in ECG‐measured TpTe (Cluitmans et al., 2021). However, our findings are consistent with previous experimental (Hsieh et al., 2014; Wisløff‐Aase et al., 2020) and clinical findings (Kim et al., 2014; Thomsen et al., 2021) at moderate hypothermia. Experimental *in vitro* models have demonstrated a profound increase in dispersions of repolarization at temperatures <30°C, but a remarkable shift at temperatures ≥32°C with reduced dispersion compared to lower temperatures, and an arrhythmogenicity similar to 36°C (Hsieh et al., 2014; Piktel et al., 2011). In ischemic experimental models, a direct temperature‐related beneficial attenuation of arrhythmic susceptibility has been observed ≥32°C, due to a reduction in both dispersion of repolarization and conduction‐slowing (Nassal et al., 2017; Piktel et al., 2012). After rewarming we found that dispersion of repolarization was slightly increased compared to T1 at spontaneous and comparable HR. We carefully suggest that these findings probably represent myocardial stunning after cold ischemia between the measurements at T2 and T3, and not a pro‐arrhythmic change due to cooling to 32°C.

In patients with coronary artery disease, and after myocardial infarction, mechanical dispersion is frequently enhanced due to heterogeneity between healthy and dyskinetic myocardial tissue, with further increased arrhythmogenicity (Haugaa et al., 2013; Stankovic et al., 2015). At 32°C, myocardial contraction in our study was prolonged. However mechanical dispersion remained unchanged at spontaneous and comparable HR. This novel finding indicates that moderate hypothermia does not exaggerate mechanical heterogeneity. After rewarming myocardial contraction remained prolonged at spontaneous HR although not during pacing. Time to peak systolic strain increased but IVCT and ejection time were not significantly prolonged, despite an apparent trend. The possible presence of myocardial stunning combined with increased HR 90 bpm at T3, may explain these divergent myocardial responses.

The isolated impact of moderate hypothermia on electrophysiological and mechanical relationships in our study was increased electromechanical window positivity. Despite the different physiological stimulus compared to long QT syndrome and patients with cardiac disease, we carefully suggest that this finding may have clinical relevance. Measuring the electromechanical window in clinical practice can provide valuable information when treating patients with temperature control, and when deciding the optimal individual protocol in comatose cardiac arrest survivors, according to the recently revised guidelines.

### 4.1 Limitations

The study has some confounders. The open thorax and the surgical setting with changes in autonomic tone and catecholamine levels, may influence electrical changes and mechanical performance (Boudoulas et al., 1981; Skulstad et al., 2004). However, these aspects were compensated by the advantageous clinical study design analogous an experimental model. All measurements were performed during comparable standardized conditions and with comparable HR at all temperatures. Thus, each patient acted as its own control. Yet, it must be emphasized that the registrations at T3 were performed after completed graft surgery which may have induced myocardial stunning. The aim of the study was to elucidate the isolated effects of moderate hypothermia in a population without overt cardiac disease. However, the findings could prove highly relevant for the clinical assessment of hypothermic patients, and especially comatose cardiac arrest survivors with a non‐cardiac cause of the arrest.

## 5 CONCLUSIONS

In patients with normal left ventricular function, electrical and mechanical systole were prolonged during moderate hypothermia without exaggerating electrical and mechanical dispersion. Electromechanical window positivity increased which is associated with decreased arrhythmic susceptibility in other clinical conditions. This temperature‐related impact may be clinically relevant in selected groups of patients after cardiac arrest.

## CONFLICT OF INTEREST

The authors declare that there is no conflict of interest and no financial disclosure.

## AUTHOR CONTRIBUTIONS

All authors have contributed substantially in conducting the underlying research and drafting this manuscript. All authors read and approved the final manuscript.

## ETHICS APPROVAL

Ethics approval and consent to participate: yes; consent for publication: yes.
